# Abstracts

**DOI:** 10.1155/S1463924685000256

**Published:** 1985


Journal of Automatic Chemistry/*Journal of Clinical Laboratory Automation*, Vol. 7, No. 3 (Jul–Sep 1985), pp. 121–123

### Abstracts

### Page 124

### Logarithms and exponentiation in MUMPS: routines for the clinical biochemistry laboratory computer system

T. G. Pellar and A. R. Henderson

The MUMPS language does not include the arithmetic functions for logarithms or exponentiation; neither does it have an exponentiation operator. These deficiencies limit its use for some scientific applications requiring these capabilities. We have written a set of routines in MUMPS that provide logarithmic and exponentiation (antilogarithms) and which can be used like our calculator routines (*Journal of Automatic Chemistry*, 7 [April–June 1985], p. 95). The routines require about 9·95 kbyte of core memory.

### Logarithmes et exponentiation avec MUMPS: Routines pour le système d'ordinateur pour le laboratoire de biochemie clinique

T. G. Pellar and A. R. Henderson

Le langage MUMPS n'inclut pas les fonctions arithmétiques pour logarithmes ou exponentiation; il ne contient non plus un opérateur d'exponentiation. Ceci limite son utilisation pour certaines applications scientifiques qui requièrent ces capabilités. Nous avons écrit un nombre de routines en MUMPS qui produisent des logarithmes et l'exponentiation (antilogarithmes) et qui peuvent être utilisées comme une routine de calculateur (*Journal of Automatic Chemistry*, 7, [1985], p. 95). Ces routines occupent environ 9·95 kbyte de mémoire centrale.

### Logarithmen-und Exponential-Funktionen in MUMPS: Programme für das klinisch-biochemische Laborcomputer system

T. G. Pellar and A. R. Henderson

Die MUMPS Sprache umfasst keine Logarithmischen-und Exponential-Funktionen; auch gibt es keinen Exponential-Operator. Dieser Mangel schränkt die Benützung für gewisse wissenschaftliche Applikationen ein, die diese Eigenschaften erfordern. Wir haben einen Satz von Programmen in MUMPS geschrieben, der Logarithmische und Exponential-Funktionen (Antilogarithmen) umfasst und der wie unsere Rechenoperations-Programme (*Journal of Automatic Chemistry*, 7 [1985], p. 95) benützt werden kann. Die Programme benötigen ca. 9,95 kbyte Speicherplatz.

---

### Page 130

### Automation in multi-dimensional gas chromatography

G. M.Ogle *et al.*

The development of the microprocessor and its incorporation into analytical instrumentation has led to a great many improvements in performance. These improvements are illustrated by looking at the field of gas chromatography.

Multi-dimensional gas chromatography is a technique whereby more than one column is used in the analytical system leading to very high resolution of the components in the sample. Many requirements are placed on the instrumentation by the use of multi-dimensional chromatography and these are illustrated by the design features of a recently released gas chromatograph.

A multi-dimensional 'heart cut' system is described in detail and its application to the analysis of alcohols in petrol is discussed. Due to environmental pressures, the use of lead alkyls as anti-'knock' ingredients in fuel is becoming less common and the low alcohols from $C_2$ to $C_5$ are being used increasingly. Using multi-dimensional chromatography, the analysis time is approximately 10 min instead of 1 h when using a single capillary column, leading to a significant increase in the number of samples analysed in a day.

French and Germans abstracts will be published in our next issue.

---

### Page 136

### Laboratory Information Management Systems—a survey

K. Jones

Recently, laboratory information management systems have received widespread attention in the UK and elsewhere. As yet there has been no independent survey of the market needs or potential. A survey, the results or which are published here, has been carried out as part of the requirement for a business degree at the Manchester Business School. This survey attempts to determine the present status of analytical laboratories in the UK in terms of the potential for the installation of LIM systems.

French and German abstracts will be in No. 4.

---

### Page 141

### The design of dilution loops for continuous-flow analysis

S. C. Coverly

The design of dilution loops for various applications on the Auto-Analyzer II is discussed, and recently developed techniques are described, including those allowing segmentation of the resampled flow. Examples of alternative approaches to a ten-fold dilution are given, and the theoretical dispersion calculated. The comparative merits of dilution loops and dialysers are discussed briefly.

**Une construction de boucles de dilution pour l'analyse à flux continu**
S. C. Coverly

La construction de boucles de dilution pour différentes applications avec le AutoAnalyzer II est discutée, les techniques développées récemment sont décrites incluant celles permettant la segmentation du flux rééchantillonné. Des examples d'une alternative à une dilution du facteur 10 sont donnés et la dispersion théorique est calculée. Les mérites comparatifs de boucles de dilution et de dialyseurs sont brièvement discutés.

**Die Konstruktion von Verdünnungsschlaufen für die kontinuierliche Durchflussanalyse**

S. C. Coverly

Es wird die Konstruktion von Verdünnungsschlaufen für verschiedene Anwendungen auf dem Auto-Analyzer II diskutiert. Es werden auch kürzlich entwickelte Techniken beschrieben, umfassend auch solche die eine Segmentierung des erneuten Probenflusses erlauben. Es werden Beispiele von Alternativen für eine 10-fache Verdünnung gegeben, und es wird die theoretische Dispersion berechnet. Die vergleichbaren Vorzüge von Verdünnungsschlaufen und Dialysatoren werden kurz diskutiert.

---

### Page 145

**Continuous-flow analysis: the Auto-Analyzer**
C. Crandell

In an effort to maximize the throughput of samples and reduce the tedium of repetitive analyses in clinical and analytical laboratories, continuous-flow analysis (CFA) was developed. This technique has been introduced commercially under the name Auto-Analyzer. Depending upon operating conditions, a number of problems can crop up, and further, calculations and report formats vary from user to user. These problems are addressed, along with how a programmable computing integrator can be used to solve them.

**Analyses à flux continu: l'"Auto-Analyzeur'**

C. Crandell

En vue de maximiser le nombre d'échantillons et de réduire la peine des analyses répétitives en laboratoires cliniques et analytiques, l'analyse continu (CFA) a été développée. Cette technique a été introduite commercialement sous le nom de 'Auto-Analyzer'. Dépendant des conditions d'opération un nombre de problèmes peut apparaître, et les calculs et les formes des comptes-rendu peuvent varier d'un utilisateur à l'autre. Ces problèmes sont présentés ainsi qu'une solution possible à l'aide d'un intégrateur programmable.

**Kontinuierliche Durchflussanalyse: Der Auto-Analyzer**
C. Crandell

Im Bemühen um die Erhöhung des Probendurchsatzes und der Reduktion der Mühen repetitive Analysen in klinischen und analytischen Laboratorien wurde die kontinuierliche Durchflussanalyse entwickelt. Diese Technik wurde kommerziell auf dem Markt unter dem Namen Auto-Analyzer eingeführt. Je nach Betriebsbedingungen können eine Reihe von Problemen auftreten. Zudem variieren Berechnungen und Formatierung des Analysenberichts von Benützer zu Benützer. Diese Probleme werden angesprochen, zusammen mit einer Beschreibung wie programmierbare Rechenintegratoren für die Lösung benützt werden können.

---

### Page 149

**A modification of the Hilger Analytical Chemispek multichannel electrolyte analyser for the direct measurement of urine concentrations of sodium, potassium, urea and creatinine**
D. A. Mallinson

A modification of the Hilger Analytical Chemispek multichannel electrolyte analyser for the measurement and print-out of the concentrations of sodium, potassium, urea and creatinine by direct sampling of undiluted urine is described. The results correlated well both with those produced on the same instrument using manual dilution of urine for potassium, urea and creatinine and also with those produced on the Beckman Astra-4 using manual dilution for urea only. Imprecision was similar for all the methods.

Operator time is less than half that required with the other methods; reagent costs are half those of the original method and less than 10% of those of the Astra-4 using manufacturer's reagents.

**Une modification de l'analyseur d'électrolytes Hilger Analytical Chemispek à canaux multiples pour la mesure directe des concentrations de sodium, potassium, urée et créatinine dans l'urine**
D. A. Mallinson

Une modification de l'analyseur d'électrolytes Hilger Analytical Chemispek à canaux multiples pour la mesure et le compte rendu des concentrations de sodium, potassium, urée et créatinine par échantillonnage direct d'urine non-diluée est décrite. Les résultats montrent une belle corrélation avec ceux produits sur le même instrument utilisant la dilution manuelle de l'urine pour le potassium, l'urée et la créatinine et également avec les résultats produits par le Beckman Astra-4 utilisant la dilution manuelle pour l'urée. L'imprécision était comparable pour toutes les méthodes.

Le temps d'opérateur est diminué de moitié comparé aux autres méthodes; les coûts de réactif sont de moitié comparés à la méthode originale et moins d'un dixième comparé à ceux de l'Astra-4 utilisant les réactifs du manufacturier.

**Eine Modifikation des Hilger Analytical Chemispek Vielkanal-Elektrolytanalysators für die direkte Bestimmung von Natrium, Kalium, Harnstoff und Kreatinin in Urin**
D. A. Mallinson

Es wird eine Modifikation des Hilger Analytical Chemispek Vielkanal-

Elektrolytanalysators für Messung und Ausdruck der Konzentrationen von Natrium, Kalium, Harnstoff und Kreatinin in unverdünnten Proben von Urin beschrieben. Die Resultate korrelierten gut mit denen, die auf dem gleichen Instrument für die Messung von Kalium, Harnstoff- und Kreatinin in manuell verdünntem Urin erhalten wurden, als auch mit den Resultaten, die mit einem Beckman Astra-4 unter Benützung einer manuellen Verdünnung für Harnstoff erzielt wurden. Die Ungefür nauigkeit war für alle Methoden etwa die gleiche.

Der Benützerzeitaufwand ist weniger als die Hälfte dessen, was bei den anderen Methoden erforderlich ist; die Kosten der Reagenzien betragen die Hälfte der ursprünglichen Methode und weniger als einen Zehntel im Vergleich zur Benützung des Astra-4 bei Verwendung der Reagenzien des Herstellers.

---

### Page 152

### An automated determination of beta-glucuronidase activity in human serum with the Abbott VP bichromatic analyser

C. G. Bao and F. V. Alvarez

This is the description of an automated method for determining the enzymatic activity of b-glucuronidase with an Abbott VP Bichromatic Analyzer. The method is linear to 110 IU/l, demonstrating a CVs of (between-day) 3.48 and (within-run) 1.23. This automated method reduces the time required for an enzymatic assay to less than 25% of that of the other methods employed in the determination of b-glucuronidase activity. Using the same quantities of reagents as the reference method, the automated method yields a considerably greater number of enzymatic assays, thus proving significantly more economical. Its correlation with the reference method is excellent. The regression equation is:

$y = 1{\cdot}047x - 0{\cdot}238$ $(N = 40)$ $(r = 0.978)$

The normal values obtained by this method are as follows: male 0·5–2·6 IU/l; female 0·5–1·8 IU/l.

### Détermination automatique de l'activité de la beta-glucuronidase dans le sérum humain avec l'analyseur Abbott VP Bichromatic

C. G. Bao and F. V. Alvarez

Ceci est une description d'une méthode automatisée pour déterminer l'activité enzymatique de la beta-glucuronidase avec un Abbott VP Bichromatic Analyzer. La méthode est linéaire jusqu'à 110 unités internationales par litre (UI/l) et un coéfficient de variation (de jour à jour) de 3.48 et (dans la même série) 1.23.

Cette méthode automatique réduit le temps réquis pour un dosage enzymatique à moins d'un quart de celui nécessité par d'autres méthodes pour la détermination de l'activité de la beta- glucuronidase. Utilisant la même quantité de réactif que la méthode de référence, la méthode automatique permet un nombre considérablement plus grand de dosages enzymatiques, et donc est nettement plus économique. La corrélation avec la méthode de référence est excellente. L'équation de regression:

$y = 1{\cdot}047x - 0{\cdot}238$ $(N = 40)$ $(r = 0{\cdot}978)$

Les valeurs normales obtenues par cettes méthode sont les suivantes: hommes 0·5–2·6 UI/l; femmes 0·5–1·8 UI/l.

### Eine automatisierte Bestimmung von Beta-Glucuronidase-Aktivität in menschlichem Serum mit dem Abbot VP Bichromat-Analysengerät

C. G. Bao and F. V. Alvarez

Es wird eine automatisierte Methode für die Bestimmung der Enzymaktivität von Beta-Glucuronidase mit dem Abbott VP Bichromat-Analysengerät beschrieben. Die Methode ist bis zu 110 IU/l linear und demonstriert einen CVS von. 3.48 (von Tag zu Tag) und von 1.23 (innerhalb einer Serie).

Diese automatisierte Methode reduziert den erforderlichen Zeitaufwand für eine enzymatische Analyse auf weniger als einen Viertel im Vergleich zu den anderen Methoden, die für die Bestimmung von Beta-Glucuronidase-Aktivität verwendet werden. Bei gleichen Vergleich an Reagenzien wie die Referenzmethoden erlaubt die automatisierte Methode eine beträchtlich grössere Anzahl von Analysen und ist deshalb bedeutend wirtschaftlicher. Die Korrelation mit der Referenzmethode ist bestens. Die Regressions-gleich ist:

$y = 1{\cdot}047x - 0{\cdot}238$ $(N = 40)$ $(r = 0{\cdot}978)$

Die Normalwerte, die mit dieser Methode erhalten werden, sind die folgenden: Männer 0·5–2·6 IU/l; Frauen 0·5–1·8 IU/l.

---

### Page 155

### A microscope stage controlled by a BBC Model B microcomputer

M. Nieman *et al*

A manually operated microscope stage has been adapted for operation by stepping motors. These are controlled by a BBC Model B microcomputer in a low-cost flexible system which allows specimens to be studied with a low risk of contamination.

### Un plateau de microscope contrôlé par un microordinateur BBC modèle B

M. Nieman *et al.*

Un plateau de microscope opéré manuellement a été adapté pour opération par moteurs pas-a-pas. Ceux-ci sont contrôlés par un microordinateur BBC modèle B dans un système économique et flexible qui permet l'étude de spécimens avec un faible risque de contamination.

### Ein Mikroskoptisch gesteurert von einem BBC Mikrocomputer Modell B

M. Nieman *et al.*

Ein manuell betriebener Mikroskoptisch wurde eingerichtet für den Betrieb mit Schrittmotoren. Diese werden von einem BBC Mikrocomputer Modell B in einem kostengünstigen, flexiblen System gesteuert, welches erlaubt Proben mit einem geringen Risiko von Verschmutzung zu studieren.

